# Circular RNAs: Functions and Clinical Significance in Cardiovascular Disease

**DOI:** 10.3389/fcell.2020.584051

**Published:** 2020-09-29

**Authors:** Lei Zhang, Yuan Zhang, Yin Wang, Yanfang Zhao, Han Ding, Peifeng Li

**Affiliations:** ^1^Institute for Translational Medicine, The Affiliated Hospital of Qingdao University, Qingdao University, Qingdao, China; ^2^Institute of Biomedical Research, School for Life Science, Shandong University of Technology, Zibo, China

**Keywords:** cardiovascular disease, circular RNAs, pathogenesis, diagnosis, clinical application

## Abstract

Cardiovascular disease (CVD) causes high morbidity and mortality worldwide. Accumulating research has indicated the possible roles played by circular RNAs (circRNAs) in the pathogenesis of CVD. CircRNAs are non-coding RNAs with covalently closed loop structures. CircRNAs can function by acting as miRNA sponges, RNA binding protein sponges, mRNA transcriptional regulators and templates for protein translation. The specific characteristics of circRNAs such as high stability, abundant distribution, and tissue- and developmental stage-specific expression make them potential biomarkers for the diagnosis and prognosis of CVD. In this paper, we systematically summarized the current knowledge regarding the biogenesis, biological properties and the action mechanisms of circRNAs, elucidated the roles played by circRNAs in the pathogenesis of CVD, and explored the diagnostic potential of circRNAs in CVD. With in-depth studies, an increasing number of molecular mechanisms underlying the participation of circRNAs in CVD may be elucidated, and the application of circRNAs in the clinical diagnosis and prevention of CVD may eventually be realized.

## Introduction

Cardiovascular disease (CVD) is one of the leading causes of morbidity and mortality worldwide. In recent decades, scientists have made considerable progress in the diagnosis and treatment of CVD. However, the increasing tendency of the mortality rate of CVD has not been stopped to date. Therefore, novel and effective strategies for the diagnostic and therapeutic interventions of CVD are strongly warranted. A growing number of studies have determined that non-coding RNAs, such as microRNAs (miRNAs) and long non-coding RNAs (lncRNAs), participate in the pathological processes of CVD and can serve as biological markers in diagnosis, prognosis and clinical treatment (Yang et al., 2016; Zhang L. et al., 2018, 2020). In the last several years, circular RNAs (circRNAs) have also been reported to be associated with CVD.

CircRNAs are covalently closed loop structures with no 5′ cap and 3′ polyadenylated tail. CircRNAs were first identified in plant viruses (Kolakofsky, 1976) and were thought to have no function or very limited function (Nigro et al., 1991; Cocquerelle et al., 1992; Capel et al., 1993). Subsequently, the existence of circRNAs has also been reported in many organisms, such as yeast (Schroeder et al., 1983) and humans (Cocquerelle et al., 1993). The rapid development of prediction, detection and screening technologies for circRNAs facilitates the discovery of different types of circRNAs. Studies have reported that circRNAs might participate in the regulation of physiological and pathological processes of different kinds of CVD (Fan et al., 2017; Lim et al., 2020), such as myocardial infarction (MI) (Geng et al., 2016; Cai et al., 2019), cardiac senescence (Du et al., 2016; Chen et al., 2018) and coronary artery disease (CAD) (Holdt et al., 2016; Dang et al., 2017; Shan et al., 2017). CircRNAs have a variety of characteristics, including high stability, tissue-and developmental-specific expression, and the altered expression in the pathological and normal conditions of various diseases (Werfel et al., 2016; Siede et al., 2017; Gupta et al., 2018). Due to these characteristics, circRNAs exhibit considerable potential as biomarkers for the detection of CVD from human blood samples (Vausort et al., 2016; Zhao et al., 2017). In this paper, we will summarize the available knowledge on the biogenesis of circRNAs, the functions of circRNAs, the roles of circRNAs in CVD and the diagnostic potential of circRNAs in CVD.

## Biogenesis of CircRNAs

**FIGURE 1:**
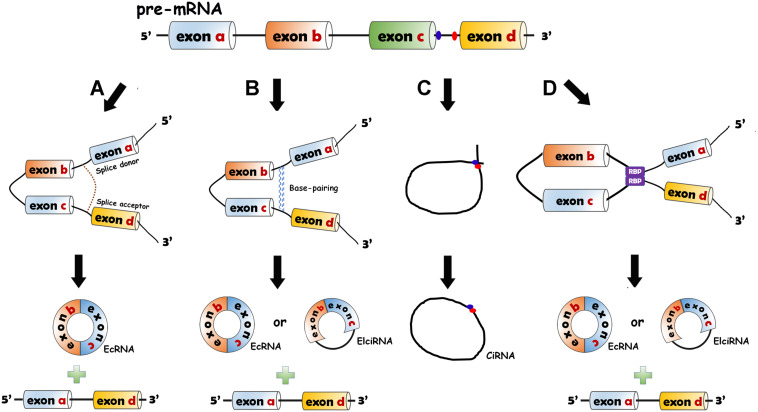
Biogenesis models of circRNAs. **(A)** Lariat-driven circularization model. The 5′ end of the intron (splice donor site, GU) and the 3′ end of the intron (splice acceptor site, AG) are covalently bound to generate an exon-containing lariat (one or more exons) which is internally spliced to form EcRNA. **(B)** Intron-pairing-driven circularization model. The RNA base motifs (e.g., Alu repeats) in the introns of pre-mRNA pair with the complementary sequences and the direct cyclization happens to generate ecRNAs or exon–intron circRNAs (EIciRNAs). **(C)** Circular intronic RNA (ciRNA) formation. The intron lariat is produced by backsplicing process. C-rich element close to the branch and the GU-rich element near the 5′ splice site bind together. The other exons and introns are removed by the spliceosome. **(D)** RNA binding protein (RBP)-driven model. The bridging of RBPs with pre-mRNAs can give rise to the production of circRNAs (ecRNAs or EIciRNAs).

CircRNAs are divided into three categories: exonic circRNAs (ecircRNAs or ecRNAs) (Zhang et al., 2014), circular intronic RNAs (ciRNAs) (Zhang et al., 2013) and exon–intron circRNAs (EIciRNAs) (Li et al., 2015). CircRNAs are generated from pre-mRNAs through backsplicing. Four mechanisms of circRNA formation have been revealed. The 5′ end of the intron (splice donor site, GU) and the 3′ end of the intron (splice acceptor site, AG) can be covalently bound to generate an exon-containing lariat, which will be internally spliced thereafter to form an exonic circle (Jeck et al., 2013; Jeck and Sharpless, 2014) (Figure 1A). The RNA base motifs (e.g., Alu repeats) in the introns of pre-mRNA can pair with the complementary sequences (Jeck et al., 2013; Zhang et al., 2014), and direct cyclization subsequently occurs to generate ecRNAs (introns removed) or EIciRNAs (introns retained) (Jeck et al., 2013) (Figure 1B). In the introns, the C-rich element close to the branch and the GU-rich element close to the 5′ splice site can bind together, and then the other exons and introns are removed by the spliceosome to form ciRNAs (Zhang et al., 2013) (Figure 1C). The bridging of RNA binding proteins (RBPs) with pre-mRNAs has also been elaborated to facilitate the production of circRNAs (ecRNAs or EIciRNAs) (Ashwal-Fluss et al., 2014; Conn et al., 2015) (Figure 1D).

Multiple factors participate in the biogenesis of circRNAs. Quaking (QKI) and Muscleblind (MBL) proteins function as regulatory activators of the biogenesis of circRNAs (Ashwal-Fluss et al., 2014; Conn et al., 2015). In contrast, an adenosine deaminase acting on RNA-1 inhibits the circRNA formation by destroying the stem structures (Rybak-Wolf et al., 2015). Serine–arginine and heterogeneous nuclear ribonucleoprotein can regulate the generation of circRNAs in Drosophila (Kramer et al., 2015).

## Biological Properties of CircRNAs

CircRNAs have some common characteristics and the most important ones are elaborated as follows.

(1) Wide distribution and diversity. CircRNAs are found in many eukaryotic organisms ranging from plants to animals and in all tissues (Jeck et al., 2013). In humans, over 30,000 circRNAs have been found and the number will increase in the future (Xu et al., 2017; Zeng X. X. et al., 2017).
(2) High stability. Because of the covalently closed structures, circRNAs are resistant to the degradation by ribonuclease (RNase) and are more stable than linear RNAs (Suzuki et al., 2006).
(3) Specific expression. CircRNAs are specifically expressed in different tissues, cells and developmental stages (Jakobi et al., 2016; Li Y. S. et al., 2017; Xu et al., 2017). circRNAs have different profiles at four stages of heart differentiation (Li Y. S. et al., 2017). Significant alterations have been detected at different developmental stages in cardiomyocytes derived from induced pluripotent stem cells (Siede et al., 2017).
(4) Evolutionary conservation. Many circRNAs seem to be evolutionarily conserved among species (Jeck et al., 2013; AbouHaidar et al., 2014). Jeck et al. (2013) found the homology of 2121 circRNAs between human fibroblasts and mouse genome. Werfel et al. (2016) reported high homology of 1288 circRNAs across human, mouse and rat. However, Werfel et al. (2016) also revealed that only a small number of circRNAs were conserved. Other studies have also illustrated that many circRNAs are specific to species (Aufiero et al., 2019; Lim et al., 2020).
(5) Dynamic expression profiles between normal and pathological conditions. A lot of circRNAs have altered expression related to diseases. Zheng et al. (2016) revealed altered expression of many circRNAs between normal tissues and cancerous tissues. In many other diseases, the expression differences of circRNAs have also been verified (Werfel et al., 2016; Siede et al., 2017; Gupta et al., 2018). Some studies reported lack of dynamic expression of circRNAs in specific diseases (Werfel et al., 2016; Tan et al., 2017).

## The Underlying Mechanisms of CircRNA Functions

**FIGURE 2:**
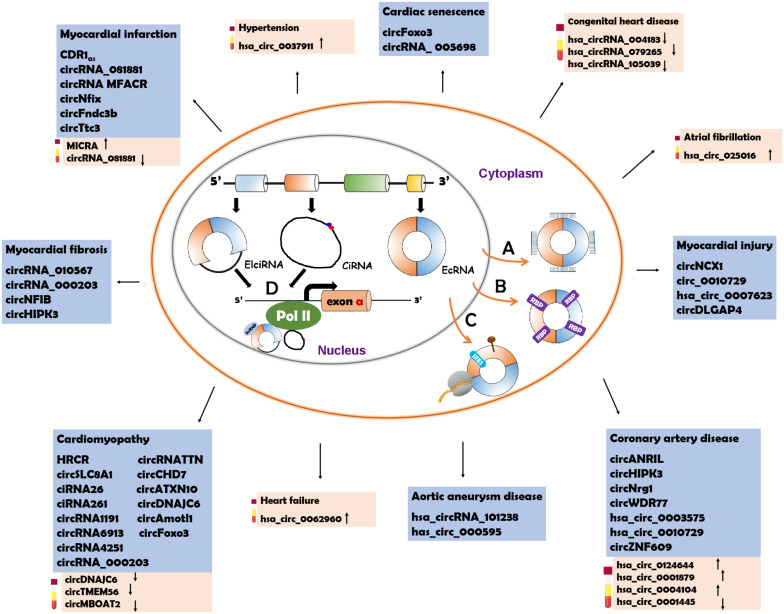
Underlying action mechanisms of circRNAs and the circRNAs relevant to CVD pathogenesis. **(A)** CircRNAs can serve as miRNA sponges to regulate the functions of miRNAs. **(B)** CircRNAs can bind to RNA binding proteins (RBPs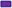) to influence their functions. **(C)** CircRNAs can be the templates to encode proteins with the help of internal ribosome entry site (IRES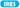) elements or m^6^A-modification (brown pin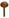). **(D)** circRNAs with intronic sequences can regulate the expression of parental genes through binding to RNA polymerase II (Pol II). The circRNAs involved in CVD are listed in the blue box. The possible diagnostic biomarkers are displayed in the pink box. EcRNA, exonic circRNA; EIciRNA, exon–intron circRNA; CiRNA, circular intronic RNA.

The characteristics of circRNAs, such as wide distribution, high stability, expression specificity and localization, indicate that circRNAs have various biological functions. Recent studies have illustrated that circRNAs can function through different mechanisms (Figure 2).

### Some CircRNAs Act as miRNA Sponges

CircRNAs contain miRNA response elements (MREs) that facilitate the binding of circRNAs and miRNAs. This binding decreases the level of functional miRNAs and thus increases the expression of miRNA targets (Hansen et al., 2013a; Tay et al., 2014). This process is known as the “sponge effect,” as circRNAs can absorb miRNAs similar to sponges (Figure 2A). Many circRNAs can function as miRNA sponges. CiRS-7/CDR1_*as*_ contains more than 70 binding sites for miR-7 that are involved in the pathogenesis of various diseases (Hansen et al., 2013b; Pan et al., 2018; Liu et al., 2019). The binding of ciRS-7/CDR1as to miR-7 causes downregulation of miR-7 and elevated levels of miR-7 target genes. Sry, a testis-specific circRNA, has 16 conserved MREs for miR-138 (Hansen et al., 2013a). The activity of miR-138 is inhibited due to the sponge effect and the miR-138 target genes are upregulated (Hansen et al., 2013a). CircHIPK3 have different MREs (total number: 18) which allow it to bind to nine different miRNAs (Zheng et al., 2016). HRCR can bind to miR-223 which promotes the pathogenesis of cardiac hypertrophy and heart failure (HF) (Wang et al., 2016). CircNFIB can competitively absorb miR-433 to enhance cardiac fibroblast proliferation induced by the stimulation of TGF-β (Zhu et al., 2019). CircRNA_000203 can directly sponge miR-26b-5p and miR-140-3p to regulate the occurrence of cardiac hypertrophy (Li et al., 2019).

### Some CircRNAs Serve as RBP Sponges

CircRNAs can interact with RBPs and inhibit RBP activity (Figure 2B). CircMbl is produced from the same pre-mRNA as the MBL protein and can absorb MBL proteins to regulate the subsequent physiological processes (Ashwal-Fluss et al., 2014). CircPABPN1 can bind to HuR, which is a well-known RBP, to prevent the interaction between HuR and PABPN1 mRNA, suppressing the translation of PABPN1 mRNA (Abdelmohsen et al., 2017). CircANRIL competitively recruits PES1 (an essential 60S-preribosomal assembly factor), leading to inhibition of ribosome biogenesis (Holdt et al., 2016). CircFoxo3 participates in the processes of cardiomyocyte senescence and cell cycle progression through interacting with different RBPs, such as anti-senescent protein ID-1, transcription factor E2F1, anti-stress proteins FAK, p21 (cyclin-dependent kinase inhibitor 1) and CDK2 (cyclin-dependent kinase 2) (Du et al., 2017). CircAmotl1 can protect cardiomyocytes and promote cell proliferation and wound repair by binding to the cardioprotective molecules (PDK1 and AKT1) and to STAT3 (signal transducer and activator of transcription 3) (Yang Z. G. et al., 2017; Zeng Y. et al., 2017).

### Some CircRNAs Encode Peptides

Studies conducted in recent years have demonstrated that circRNAs can serve as templates for protein translation (Chen and Sarnow, 1995; Wesselhoeft et al., 2018) (Figure 2C). It was first observed in prokaryotes that circRNAs were able to encode proteins. In 1986, studies in hepatitis D virus showed that circRNAs could be translated into functional proteins (Kos et al., 1986). In *Escherichia coli*, a circRNA was also reported to act as a translation template (Perriman and Ares, 1998). CircRNAs lack a 5′ cap and a 3′ polyadenylated tail which are typical translation initiation structures of linear RNAs. Nevertheless, instead of recruiting ribosomes, circRNA translation is initiated with the help of specific elements, such as IRES (internal ribosome entry site) and *N*-methyladenosine (m^6^A) (Chen and Sarnow, 1995; Wesselhoeft et al., 2018). When IRESs are introduced into a circRNA, the synthetic circRNA initiates translation (Chen and Sarnow, 1995). CircZNF609 has an IRES element and can be translated into a protein that functions in myoblast proliferation (Legnini et al., 2017). CircFBXW7 can be translated into a functional protein that plays a role in the inhibition of glioma tumorigenesis with the assistance of the IRES element (Yang et al., 2018). m^6^A modification has been illustrated to facilitate the translation of linear mRNAs in a cap-independent manner (Meyer et al., 2015). Some circRNAs also employ m^6^A to initiate translation (Yang Y. et al., 2017). The m^6^A reader YTHDF3 can interact with circRNAs and subsequently recruit translation initiation factors to drive the initiation of circRNA translation (Yang Y. et al., 2017). In a study based on ribosome profiling of Drosophila heads, circMbl3 was demonstrated to be the template for splicing-dependent translation (Pamudurti et al., 2017). Another recent study found that circβ-catenin could be translated into the β-CATENIN isoform, a functional protein that activates the Wnt/β-catenin pathway and promotes tumor development in liver cancer (Liang et al., 2019).

### Some CircRNAs Regulate the Transcription of Parental Genes

Of the three types of circRNAs, ecRNAs account for the majority and are predominantly located in the cytosol (Hansen et al., 2013a; Memczak et al., 2013). The cytoplasmic localization of ecRNAs facilitates their functions as miRNA sponges, RBP sponges and translational templates. CiRNAs and EIciRNAs are confined to the nucleus due to their intronic sequences (Zhang et al., 2013). Recent studies have verified the roles of nucleus-localized ciRNA and EIciRNA in the transcriptional regulation of their parental genes (Zhang et al., 2013; Li et al., 2015) (Figure 2D). CiRNAs can directly interact with RNA polymerase II (Pol II) and enhance parental gene transcription (Zhang et al., 2013), whereas EIciRNAs bind to the U1 small nuclear ribonucleoproteins (snRNPs) at first, and the complex promotes the interaction with Pol II (Li et al., 2015). CiRNA-ankrd52 can bind to Pol II of the pre-mRNA of the *ANKRD52* gene to promote transcription, while the knockdown of ciankrd52 may cause a significant transcriptional decrease in the parental gene (Zhang et al., 2013). CircEIF3J and circPAIP2 are EIciRNAs that can form a complex with U1 snRNP and Pol II (Li et al., 2015). In general, ciRNAs and EIciRNAs can serve as transcription regulators of their parental genes.

## CircRNA Identification and Research Databases

Various methods have been developed to identify and study the functions of circRNAs. RNA-seq, microarray, Northern Blot and RT-PCR analyses are the most widely used. RNA-seq can identify new circRNA species and quantify circRNA expression. Next-generation sequencing and depletion of ribosomal RNA methods may be cooperatively utilized for circRNA-seq. The microarray method can only detect and quantify known circRNAs approximately and the results require further confirmation. For high-throughout sequencing, reliable and precise algorithms are of vital importance. Many algorithms have been developed, such as circRNA-finder, MapSplice, CIRCexplorer, and CIRI (Hansen et al., 2016). The combined use of these algorithms may improve the accuracy and sensitivity of circRNA identification. Northern Blot and RT-PCR methods only validate known circRNAs dependent on an exoribonuclease-RNase R, which cleaves linear RNAs from the 3′ end (Asha et al., 1983). With circular structures, circRNAs can avoid the cleavage of RNase R. Thus, in the extraction process, total RNA is digested by RNase R to discard the linear RNAs (Szabo and Salzman, 2016). The collected RNAs are then combined with specific probes (Northern Blot method) or reverse-transcribed to obtain cDNAs of circRNAs (RT-PCR method). Aside from these methods for circRNA identification, some other methods are employed to analyze the subcellular localization (fluorescence *in situ* hybridization) or the interaction between circRNAs and miRNAs/RBPs (e.g., RNA immunoprecipitation and dual luciferase reporter assay) (Li Y. et al., 2018; Zirkel and Papantonis, 2018). In addition to these known methods, simpler and more efficient methods are still urgently needed for the identification and characterization of circRNAs.

To elucidate the roles of circRNAs, many online databases have been developed (Table 1). Different databases emphasize on different aspects of circRNA research including prediction, identification, protein-coding annotation, circRNA-miRNA interactions and circRNA-RBP interactions. The combined use of these databases may help to establish a foundation for the further studies of circRNAs.

**TABLE 1:** Database for circRNA research.

**Name**	**URL**	**Function**
**circBase**	http://www.circbase.org/	Collects the circular RNA information of many species, such as human, mouse, *C. elegans*, etc.
**CircInteractome**	https://circinteractome.nia.nih.gov/	Maps RBP- and miRNA-binding sites on human circRNAs; Designs primers and siRNA sequence of circRNAs.
**CircNet**	http://circnet.mbc.nctu.edu.tw/	Integrates the following information: identification of new circRNAs; genome annotation of circRNAs; the expression profile of circRNAs; the network of circRNA-miRNA -mRNA.
**circRNABase**	http://starbase.sysu.edu.cn/mirCircRNA.php	Integrates the published data to construct the interaction network of miRNA and circRNA, or circRNA and RBP.
**circRNADb**	http://reprod.njmu.edu.cn/circrnadb	Summarizes circRNAs that encode proteins; contains 32,914 human circRNAs.
**Circ2Traits**	http://gyanxet-beta.com/circdb/	Collects circRNAs potentially associated with human diseases or traits.
**CIRCpedia**	http://www.picb.ac.cn/rnomics/circpedia/	Provides annotations on circRNAs and variable shear events of different cell lines/tissues
**Deepbase**	http://deepbase.sysu.edu.cn/	Emphasizes the interaction of ceRNA molecular network; integrates the information related to circRNAs.
**TSCD**	http://gb.whu.edu.cn/TSCD/	Provides information of tissue-specific circRNAs in main tissues of human and mouse.
**circRNA disease**	http://cgga.org.cn:9091/circRNADisease/	Includes the data of the association between circRNAs and disease, the expression of circRNAs in the patients, the detection methods of circRNA
**CSCD**	http://gb.whu.edu.cn/CSCD	Collects cancer-specific circRNAs; elaborates the interaction between miRNA and circRNA, or circRNA and RBP; predicts variable splicing of related genes.

*TSCD, tissue specific circRNA database; CSCD, cancer-specific circRNA database; RBP, RNA binding protein.*

## CircRNAs and Cardiovascular Diseases

With the use of advanced technologies in sequencing and data analysis, a lot of circRNAs have been detected in human hearts and been reported to be associated with CVD (Jakobi et al., 2016; Werfel et al., 2016; Fan et al., 2017; Tan et al., 2017) (Table 2 and Figure 2). Moreover, circRNAs have been observed to have great potential as biomarkers for the diagnosis and prognosis of CVD (Vausort et al., 2016; Sonnenschein et al., 2019; Wu et al., 2019; Vilades et al., 2020) (Table 3 and Figure 2).

**TABLE 2:** CircRNAs with CVD.

**CVD type**	**CircRNAs**	**Source**	**Action mechanism**	**Regulation**	**References**
**Myocardial infarction**	CDR1_*as*_	Mouse myocardial tissue	Sponge miR-7	Up	Geng et al. (2016)
	CircRNA_081881	Blood	Sponge miR-548	Down	Deng et al. (2016)
	CircRNA MFACR	A/R and I/R mouse models	Sponge miR-652-3p	Up	Wang et al. (2017)
	CircNfix	Mouse heart section	Dual function as miR-214 sponge and inducing YBX1 degradation	Down	Huang et al. (2019)
	CircFndc3b	Mouse hearts	Interact with RBP-FUS	Down	Garikipati et al. (2019)
	CircTtc3	Rat myocardium	Sponge miR-15b-5p	Up	Cai et al. (2019)
**Myocardial fibrosis**	CircRNA_010567	Mouse myocardium, cardiac fibroblasts	Sponge miR-141	Up	Zhou and Yu (2017)
	CircRNA_000203	Mouse myocardium, cardiac fibroblasts	Sponge miR-26b-5p	Up	Tang et al. (2017)
	CircNFIB	Mouse heart tissue	Sponge miR-433	Down	Zhu et al. (2019)
	CircHIPK3	Mouse cardiac fibroblasts	Sponge miR-29b-3p	UP	Ni et al. (2019)
**Myocardial injury**	CircNCX1	Mouse cardiomyocytes	Sponge miR-133a-3p	Up	Li M. et al. (2018)
	Circ_0010729	Human cardiomyocytes	Sponge miR-145-5p	Down	Jin and Chen (2019)
	Hsa_circ_0007623	Acute ischemia mice	Sponge miR-297	Up	Zhang Q. et al. (2020)
	CircDLGAP4	Myocardial ischemia-reperfusion injury	Sponge miR-143	Up	Wang S. et al. (2019)
**Cardiomyopathy**	HRCR	Mouse cardiomyocytes	Sponge miR-223-5p	Down	Wang et al. (2016)
	CircSLC8A1	Mouse cardiomyocytes	Sponge miR-133a	—	Lim et al. (2019)
	CircRNA_000203	NMVCs	Sponge miR-26b-5p and miR-140-3p	Up	Li et al. (2019)
	CiRNA26	Mouse cardiomyocytes	Sponge several miRNAs	Down	Meng et al. (2019)
	CiRNA261	Mouse cardiomyocytes	Sponge several miRNAs	Up	Meng et al. (2019)
	CircRNA1191	Mouse cardiomyocytes	Sponge several miRNAs	Down	Meng et al. (2019)
	CircRNA6913	Mouse cardiomyocytes	Sponge several miRNAs	Up	Meng et al. (2019)
	CircRNA4251	Mouse cardiomyocytes	Sponge several miRNAs	Down	Meng et al. (2019)
	CircRNATTN 1/2/4/5	DCM patient with a heterozygous mutation in *RBM20* (E913K).	Unknown	Down	Khan et al. (2016)
	CircSLC8A1	Human dilated cardiomyopathy	Unknown	Up	Siede et al. (2017)
	CircCHD7	Human dilated cardiomyopathy	Unknown	Up	Siede et al. (2017)
	CircATXN10	Human dilated cardiomyopathy	Unknown	Up	Siede et al. (2017)
	CircDNAJC6	Human dilated cardiomyopathy	Unknown	Down	Siede et al. (2017)
	CircSLC8A1	Heart tissues from DCM patient	Unknown	Up	Lei et al. (2018)
	CircAmotl1	Human neonatal cardiac tissue	Interact with AKT and PDK1	Up	Zeng Y. et al. (2017)
	CircFoxo3	Mouse heart tissue	Interact with ID-1, E2F1, FAK, and HIF1a	Up	Du et al. (2017)
**Aortic aneurysm disease**	Hsa_circ_000595	Aortic smooth muscle cells	Sponge miR-19a	Up	Zheng et al. (2015)
	Hsa_circRNA_101238	Human aortic segments	Sponge miR-320a	Up	Zou et al. (2017)
**Cardiac senescence**	CircRNA005698	Sow cardiac muscle	Sponge seven miRNAs	Up	Chen et al. (2018)
	CircFoxo3	Mouse heart tissue	Interact with ID-1, E2F1, FAK, and HIF1a	Up	Du et al. (2017)
		Mouse cardiac fibroblast	p21 and CDK2	Up	Du et al. (2016)
**Coronary artery disease**	CircANRIL	Human peripheral blood	Interact with PES1	Down	Holdt et al. (2016)
		Different cell lines	interact with INK4/ARF	Down	Burd et al. (2010)
	CircHIPK3	Diabetic retinopathy	Sponge miR-30a-3p	Up	Shan et al. (2017)
	CircNrg1	MASMCs	Sponge miR-193b-5p	Down	Sun et al. (2019)
	CircWDR77	VSMCs	Sponge miR-124	Up	Chen et al. (2017)
	Nine circRNAs	VSMCs	Sponge miR-130a-3p	—	Pan et al. (2017)
	Hsa_circ_0003575	HUVECs	Sponge miR-9-5p and miR-199-3p	Up	Li C. Y. et al. (2017)
	Hsa_circ_0010729	HUVECs	Sponge miR-186	Up	Dang et al. (2017)
	CircZNF609	HUVECs	Sponge miR-615-5p	Up	Liu et al. (2017)

*VSMC, vascular smooth mother cell; SMC, smooth mother cell; PBMC, peripheral blood mononuclear cell; HUVEC, human umbilical vein endothelial cells.*

**TABLE 3:** Circulating circRNAs as diagnostic biomarkers of CVD.

**CVD type**	**CircRNAs**	**Source**	**Regulation**	**References**
**Myocardial infarction**	MICRA	Peripheral blood	Up	Vausort et al. (2016)
	CircRNA_081881	Plasma	Down	Deng et al. (2016)
**Congenital heart diseases**	Hsa_circRNA_004183	Plasma	Down	Wu et al. (2019)
	Hsa_circRNA_079265	Plasma	Down	Wu et al. (2019)
	Hsa_circRNA_105039	Plasma	Down	Wu et al. (2019)
**Hypertension**	Hsa_circ_0037911	Whole blood	Up	Bao et al. (2018)
**Cardiomyopathy**	CircDNAJC6	Serum	Down	Sonnenschein et al. (2019)
	CircTMEM56	Serum	Down	Sonnenschein et al. (2019)
	CircMBOAT2	Serum	Down	Sonnenschein et al. (2019)
**Heart failure**	Hsa_circ_0062960	Plasma	Up	Sun et al. (2020)
**Coronary artery disease**	Hsa_circ_0124644	Peripheral blood	Up	Zhao et al. (2017)
	Hsa_circ_0001879	PBMCs	Up	Wang L. et al. (2019)
	Hsa_circ_0004104	PBMCs	Up	Wang S. et al. (2019)
	Hsa_circ_0001445	Plasma	Down	Vilades et al. (2020)
**Atrial fibrillation**	Hsa_circ_025016	Plasma	Up	Zhang J. et al. (2018)

*PBMCs, peripheral blood mononuclear cells.*

### Myocardial Infarction

Myocardial infarction provokes cardiac remodeling and is often complicated by arrhythmia, shock or HF. CDR1_*as*_ has been determined to sponge miR-7 and to have elevated levels in in mice with MI (Geng et al., 2016). The increased levels of CDR1_*as*_ can upregulate PARP and SP1, the targets of miR-7, which may subsequently enlarge the size of MI (Geng et al., 2016). CircRNA_081881 has been found to be associated with acute MI. CircRNA_081881 has seven binding sites for miR-548, and the competitive binding upregulates the expression of PPARγ which can protect the heart from acute MI (Deng et al., 2016). MTP18 participates in the development of MI. CircRNA MFACR could absorb miR-652-3p to increase the expression of MTP18 and subsequently promote the progression of MI (Wang et al., 2017). CircNfix has been revealed to promote the degradation of Ybx1 and sponge miR-214. The downregulation of circNfix inhibited cardiomyocyte apoptosis after MI and promoted cardiac regeneration and repair (Huang et al., 2019). CircFndc3b exhibited significantly decreased expression levels in post-MI mouse hearts. CircFndc3b could function through interacting with RBP-FUS which regulates the expression and the signaling pathway of vascular endothelial growth factor-A (VEGF-A). The overexpression of circFndc3b in post-MI mouse hearts could decrease cardiomyocyte apoptosis and fibrosis, enhance neovascularization, reduce infarct size and attenuate left ventricular dysfunction after MI (Garikipati et al., 2019). CircTtc3 was reported to be significantly upregulated in rats with MI (Cai et al., 2019). CircTtc3 could recruit miR-15b-5p to repress its inhibitory effect on Arl2, an ADP-ribosylation factor relevant to cardiomyocyte viability (Cai et al., 2019). The knockdown of circTtc3 exacerbated the symptoms of cardiac dysfunction post-MI, suggesting that circTtc3 plays a role in the cardiac protection in MI (Cai et al., 2019).

### Myocardial Fibrosis

Myocardial fibrosis is a disease condition in which normal myocardium is replaced by non-beating cardiac fibroblasts, resulting in diastole difficulty. CircRNA_010567 exhibited significantly increased levels in both diabetic mouse myocardium and Angiotensin-II (Ang-II)-induced cardiac fibroblasts (CFs) (Zhou and Yu, 2017). CircRNA_010567 was found to recruit miR-141. The competitive binding of circRNA_010567 and miR-141 released the inhibitory effect of TGF-β1, a pro-fibrotic factor, thereby promoting myocardial fibrosis (Zhou and Yu, 2017). CircRNA_000203 was also revealed to be elevated in diabetic mouse myocardium and Ang-II-induced CFs (Tang et al., 2017). CircRNA_000203 could sponge miR-26b-5p to attenuate the inhibition of its targets, Col1a2 and CTGF, which are fibrosis-associated proteins. Thus, the upregulation of circRNA_000203 may promote the proliferation of CFs (Tang et al., 2017). CircNFIB was downregulated in primary adult CFs treated with TGF-β (Zhu et al., 2019). Overexpression of circNFIB attenuates CF proliferation while inhibition of circNFIB promotes CF proliferation, indicating the cardioprotective role of circNFIB (Zhu et al., 2019). CircHIPK3 was upregulated in CFs treated with Ang-II (Ni et al., 2019). CircHIPK3 was revealed to sponge miR-29b-3p which can target fibrosis-associated proteins such as Col1a2, Col3a1 and a-SMA. The elevated level of circHIPK3 ultimately enhanced the function of Col1a2, Col3a1 and a-SMA, thereby promoting myocardial fibrosis (Ni et al., 2019). In general, circRNA_010567, circRNA_000203 and circHIPK3 are profibrotic while circNFIB is antifibrotic.

### Myocardial Injury

Myocardial injury as well as apoptosis is usually correlated to HF, MI, and ischemia–reperfusion (I/R) injury. CircNCX1 was found to have elevated levels during oxidative stress (Li M. et al., 2018). CircNCX1 could bind to miR-133a-3p and subsequently increase the activity of cell death-inducing protein 1 (CDIP1), inducing apoptosis and I/R injury (Li M. et al., 2018). Circ_0010729 was elucidated to play a role in the injury of human cardiomyocytes induced by oxygen-glucose-deprivation (OGD) (Jin and Chen, 2019). The downregulation of circ_0010729 attenuated the OGD-induced cell injury by activating the mTOR and MEK/ERK pathways (Jin and Chen, 2019). Hsa_circ_0007623 was confirmed to have cardioprotective effects in isoproterenol-induced acute ischemia mice. Hsa_circ_0007623 was able to bind to miR-297 and repress the inhibitory effect of miR-297 on VEGF-A, thereby promoting cell proliferation, migration and angiogenesis (Zhang Q. et al., 2020). Wang S. et al. (2019) speculated that circDLGAP4 may regulate cardiomyocyte apoptosis in myocardial I/R injury through targeting miR-143. However, no additional experimental evidence has been reported to date.

### Cardiomyopathy

Cardiomyopathy is a disease with abnormal heart muscles. The muscles are stretched, weakened, or have other structural changes, causing pump difficulties of heart. Most patients with cardiomyopathy will have HF (Molkentin et al., 1998; Aaronson and Sackner-Bernstein, 2006; Rajabi et al., 2007; van Rooij et al., 2008; Authors/Task Force et al., 2014; Wang et al., 2016; Lim et al., 2019). Hypertrophic cardiomyopathy (HCM, cardiac hypertrophy) and dilated cardiomyopathy (DCM, cardiac dilatation) are two common subtypes of cardiomyopathy. When HCM happens, the heart muscles are stretched and become thick, thereby decreasing or blocking the blood flow. In DCM, the heart muscles are weakened, leading to the loss of pumping power of the heart. HRCR could bind to miR-223-5p to decrease its activity and could subsequently upregulate its target, ARC (apoptosis inhibitor with CARD domain), resulting in the inhibition of HCM and HF (Wang et al., 2016). CircSLC8A1 has recently been demonstrated to be the sponge of miR-133a (Lim et al., 2019). The knockdown of circSLC8A1 in cardiomyocytes lessened the hypertrophy induced by pressure overload, whereas the overexpression of circSLC8A1 caused HF (Lim et al., 2019). CircRNA_000203 can bind to miR-26b-5p and miR-140-3p to increase the expression level of their target gene-GATA4 (Li et al., 2019). The elevated level of GATA4 promotes the occurrence of cardiac hypertrophy (Li et al., 2019). Several circRNAs (ciRNA26, ciRNA261, circRNA1191, circRNA4251, and circRNA6913) were reported to exhibit altered expression in cardiac cells with HCM when they were cultured in high levels and normal levels of D-glucose, respectively (Meng et al., 2019). These circRNAs might be sponges of more than 60 miRNAs, suggesting that they have vital functions in HCM.

RBM20 plays a critical role in the splicing of many cardiac genes, whose mutation will cause aggressive DCM (Brauch et al., 2009). RBM20 can regulate the generation of TTN circRNA which might be involved in DCM (Khan et al., 2016). CircSLC8A1, circCHD7, and circATXN10 were found to have elevated expression levels in DCM patients compared with control patients, while circDNAJC6 expression levels were reduced (Siede et al., 2017). The increased expression level of circSLC8A1 was also observed in another study in dilated heart tissue compared with control tissues (Lei et al., 2018). CircAmotl1 has been reported to bind to PDK1 and AKT1, two cardioprotective molecules (Zeng Y. et al., 2017). This interaction activated AKT1 through phosphorylation and facilitated the nuclear translocation of AKT1 to protect cardiomyocytes in doxorubicin-induced cardiomyopathy (Zeng Y. et al., 2017). CircFoxo3 has been determined to promote doxorubicin-induced ventricular dilatation (Du et al., 2017).

### Aortic Aneurysm Disease

Aortic dissection is the most serious aneurysm disease. Through screening of aortic tissues from patients with aortic dissection aneurysms, Zheng et al. (2015) found an obviously upregulated circRNA, hsa_circ_000595. Hsa_circ_000595 was found to promote apoptosis in vascular smooth mother cells (VSMCs) under hypoxic conditions through upregulating miR-19a expression. Zou et al. (2017) found 162 circRNAs with abnormal expression by microarray analysis of three thoracic aortic dissection (TAD) patients and three control subjects, in which hsa_circRNA_101238 was notably increased. Hsa_circRNA_101238 could sponge miR-320a to inhibit its activity, thereby increasing the levels of its target, MMP9 protein (a TAD related protein) (Zou et al., 2017).

### Cardiac Senescence

Cardiac senescence greatly depresses heart function. Through high throughput RNA-seq, Chen et al. (2018) identified 22 circRNAs with dynamic expression in cardiac muscle during aging. Some of them might regulate the pro-coagulation process. CircRNA005698 was found to be a sponge for seven miRNAs and might be a biomarker for cardiac senescence (Chen et al., 2018). CircFoxo3 was reported to bind to several RBPs (ID-1, E2F1, FAK and HIF1ɑ) and inhibit their activities, thereby promoting cardiomyocyte senescence (Du et al., 2017). CircFoxo3 could also absorb two G1 to S phase transition-related proteins (p21 and CDK2) and suppress their functions in the cell cycle, leading to cell cycle repression (Du et al., 2016).

### Hypertension

Hypertension is a common chronic disease and a major risk factor for CVD. Through profiling of plasma, Wu et al. (2017) identified 59 circRNAs that exhibited altered expression between hypertensive patients and healthy controls. Additionally, a profiling with blood found 351 circRNAs that had different levels from patients with chronic thromboembolic pulmonary hypertension and healthy people (Miao et al., 2017). However, due to the small cohort (five patients and five controls for both studies), all the results in these studies require further validation. Overall, the number of studies on circRNAs in hypertension remains small. More studies should be conducted to elucidate the mechanisms.

### Coronary Artery Disease

Coronary artery disease is a chronic disease mainly caused by atherosclerosis. miRNAs have been shown to function in all pathogenesis processes of CAD (Zhang L. et al., 2018, 2020), such as endothelial dysfunction, lipid metabolism disorder, proliferation and differentiation of smooth muscle cells (SMCs). Recently, circRNAs have also been found to participate in the development of CAD. CircANRIL was testified to interact with PES1 to suppress pre-rRNA maturation and subsequently restrain the biogenesis of ribosomes, which consequently enhances the stability of anti-atherogenic cells (Holdt et al., 2016). The high level of circANRIL might reduce the severity of CAD (Holdt et al., 2016). Thus, circANRIL plays an atheroprotective role. In addition, circANRIL was also illustrated to play a role in the formation of atherosclerosis by regulating the expression of INK4/ARF (Burd et al., 2010). CircHIPK3 was found to have elevated level in diabetic retinopathy. CircHIPK3 could promote endothelial proliferation and vascular dysfunction through binding to miR-30a-3p which can target VEGFC and WNT2 (Shan et al., 2017). Neuregulin-1 (NRG1) participates in vascular physiopathology (Odiete et al., 2012). CircNrg1 was revealed to sponge miR-193b-5p which could target its host mRNA, *Nrg1*. The overexpression of circNrg1 led to an elevated level of NRG1, whereas the silencing of circNrg1 decreased the level of NRG1 (Sun et al., 2019). CircWDR77 was determined to be increased in VSMCs treated with high glucose (Chen et al., 2017). CircWDR77 could sponge miR-124 to increase the activity of its target, FGF-2 (fibroblast growth factor 2), thereby promoting VSMC proliferation and migration (Chen et al., 2017). Pan et al. (2017) identified 24 circRNAs which were differentially expressed by circRNA microarray. Among these circRNAs, nine circRNAs were found to sponge hsa-miR-130a-3p and then increase the level of TRPM3 which regulates the proliferation and contractility of VSMCs in cooperation with cholesterol (Pan et al., 2017). OxLDL treatment can be employed to induce endothelial cells injury to simulate the pathogenesis of atherosclerosis or CAD. Hsa_circ_0003575 was found to have elevated expression in oxLDL-induced HUVECs (human umbilical vein endothelial cells) (Li C. Y. et al., 2017). The study elaborated that hsa_circ_0003575 could regulate endothelial cells proliferation and angiogenesis probably through interacting with miR-9-5p and miR-199-3p (Li C. Y. et al., 2017). Dang et al. (2017) performed a circRNA microarray in hypoxia-induced HUVECs to identify 36 circRNAs with abnormal expression, and they reported that hsa_circ_0010729 was upregulated. Hsa_circ_0010729 could sponge miR-186 to regulate vascular endothelial cell proliferation and apoptosis via targeting HIF-1α. Circular RNA-ZNF609 was reported to have increased expression in HUVECs under high glucose and hypoxia stress, both *in vivo* and *in vitro*. CircZNF609 could regulate endothelial cell function by binding to miR-615-5p which targets the transcription factor MEF2A. The knockdown of circZNF609 promoted endothelial cell migration and inhibited endothelial cell apoptosis (Liu et al., 2017).

## CircRNAs as Biomarkers for CVD

Currently, a variety of circulating molecules, such as proteins and miRNAs, have been illustrated to have diagnostic potential for CVD. Such proteins as troponins, creatine kinase-MB and myoglobin have been widely used in the clinic. However, these proteins are not specific and are not applicable for the early diagnosis. Additionally, these proteins are easily influenced by such factors as the heart-associated diseases, medication, patient genetic background, and age (Chen et al., 2008; Lawrie et al., 2008). Therefore, protein biomarkers have limited diagnostic value. Circulating miRNAs have been elaborated to have high specificity and strong potential for early diagnosis. However, circulating miRNAs have not been applied in the clinic due to their low content and time-consuming detection (Zhang L. et al., 2018). Circulating circRNAs have many features resembling circulating miRNAs including high stability, sensitivity and specificity, which are essential for biomarkers. Meanwhile, the circulating levels of circRNAs are not low, and some circRNAs even have high content, making detection easier. Many studies have revealed the considerable potential of circulating circRNAs as novel and promising biomarkers for the early diagnosis of CVD (Table 3 and Figure 2).

CircZNF609 (MICRA) had lower levels in the peripheral blood of MI patients than in healthy controls (Vausort et al., 2016). Circulating MICRA was demonstrated to have a high value of predicting left ventricular dysfunction (Vausort et al., 2016). CircRNA_081881 was downregulated in the plasma of AMI patients and might be a promising target for AMI diagnosis and therapy (Deng et al., 2016). The level of hsa_circ_0124644 was increased in the peripheral blood of CAD patients and was found to have a significant association with CAD. Receiver operating characteristic (ROC) analysis revealed that circulating hsa_circ_0124644 might be a potential diagnostic biomarker for CAD (Zhao et al., 2017). Hsa_circ_0001879 and hsa_circ_0004104 were found to have increased levels in the peripheral blood mononuclear cells (PBMCs) of CAD patients (Wang L. et al., 2019). ROC analysis revealed their high accuracy in the diagnosis of CAD. Furthermore, the combination of hsa_circ_0001879, hsa_circ_0004104 and CAD risk factors had the highest value to discriminate CAD patients from healthy controls (Wang L. et al., 2019). Atrial fibrillation (AF) is a common complication after coronary artery bypass grafting (CABG) (Maesen et al., 2012). Hsa_circ_025016 was upregulated in the plasma of patients with new-onset AF after isolated off-pump CABG. ROC analysis revealed a high diagnostic value (Zhang J. et al., 2018). The analysis with a large validation cohort confirmed the diagnostic power of hsa_circ_025016 (Zhang J. et al., 2018). All results indicated that hsa_circRNA_025016 might be a promising biomarker for the prediction of new-onset AF after isolated off-pump CABG (Zhang J. et al., 2018). Sun et al. (2020) performed circRNA microarrays and found significantly upregulated plasma levels of hsa_circ_0112085, hsa_circ_0062960, hsa_circ_0053919 and hsa_circ_0014010 in HF patients. ROC analysis revealed that hsa_circ_0062960 had great potential to be a diagnostic biomarker of HF (Sun et al., 2020). A study using whole blood revealed that the hsa_circ_0037911 level was significantly increased in hypertensive patients in contrast to the control group (Bao et al., 2018). Another study revealed reduced expression levels of circRNAs (DNAJC6, TMEM56 and MBOAT2) in the serum of patients with HCM (Sonnenschein et al., 2019). All three circRNAs had high discrimination power between HCM patients and the control cohort. Moreover, circTMEM56 and circDNAJC6 could be indicators of disease severity in patients with HCM (Sonnenschein et al., 2019). Wu et al. (2019) reported three notably downregulated circRNAs (hsa_circRNA_004183, hsa_circRNA_079265 and hsa_circRNA_105039) in the plasma of children with congenital heart diseases (CHD) and employed ROC analyses to determine their potential to be biomarkers. They found the great potential of three circRNAs as novel non-invasive diagnostic biomarkers for CHD (Wu et al., 2019). Hsa_circ_0001445 was shown to have lower plasma levels in CAD patients than in the control group (Vilades et al., 2020). Hsa_circ_0001445 is secreted into circulation through being packaged in extracellular vesicles of coronary SMCs (Vilades et al., 2020). The coronary atherosclerotic condition abolished the association of hsa_circ_0001445 and vesicles, leading to the downregulation of plasma hsa_circ_0001445 (Vilades et al., 2020). Therefore, hsa_circ_0001445 might be considered an effective and novel predictor of CAD (Vilades et al., 2020). In general, these studies have illuminated the potential role of circulating circRNAs as biomarkers for the diagnosis and prognosis of CVD.

## Conclusion and Future Perspectives

Base on our exploration, the results from a variety of studies have confirmed that circRNAs can participate in the pathogenesis of CVD mainly through acting as miRNA sponges and interacting with RBPs. CircRNAs are widely distributed in different tissues and have tissue- and developmental stage-specific expression. In addition, circRNAs are stable and abundantly present in the circulatory system. Therefore, circRNAs might be promising biomarkers for the diagnosis of CVD, and accumulating research has confirmed this possibility. The clinical use of circRNAs as diagnostic biomarkers will greatly facilitate the prevention and treatment of CVD. However, there are some problems that should be solved before clinical application of circRNAs.

First, there is no generally accepted methodology on the measurement procedures of circulating circRNAs, which might result in the lack of consistency in various studies. Hence, a standardized methodology should be formulated before clinical use. Second, the sample sizes are small in most studies. The insufficient samples might lead to deviation in the test results. A larger cohort is necessary for correct conclusions. Finally, despite these findings, the underlying mechanisms of the functions of many circulating circRNAs have not been elucidated, and our knowledge is still insufficient, which represents a considerable obstacle to clinical application. More and deeper studies should be performed to explore the potential molecular mechanisms.

In summary, studies have confirmed that circRNAs are closely involved in the progression of CVD and might be promising biomarkers for CVD. These findings may provide a new avenue for the prevention, diagnosis and therapeutic intervention of CVD in the future.

## Author Contributions

LZ and YZhang drafted the manuscript. YZhao and HD edited the manuscript. YW revised the manuscript. PL and LZ conceived the idea and framework of the review and made the final proofreading. All authors read and approved the final manuscript.

## Conflict of Interest

The authors declare that the research was conducted in the absence of any commercial or financial relationships that could be construed as a potential conflict of interest.

## References

[B1] Aaronson K. D. Sackner-Bernstein J. (2006). Risk of death associated with nesiritide in patients with acutely decompensated heart failure. *JAMA* 296 1465–1466. 10.1001/jama.296.12.1465 17003394

[B2] Abdelmohsen K. Panda A. C. Munk R. Grammatikakis I. Dudekula D. B. De S. (2017). Identification of HuR target circular RNAs uncovers suppression of PABPN1 translation by CircPABPN1. *RNA Biol.* 14 361–369. 10.1080/15476286.2017.1279788 28080204 PMC5367248

[B3] AbouHaidar M. G. Venkataraman S. Golshani A. Liu B. L. Ahmad T. (2014). Novel coding, translation, and gene expression of a replicating covalently closed circular RNA of 220 nt. *Proc. Natl. Acad. Sci. U.S.A.* 111 14542–14547. 10.1073/pnas.1402814111 25253891 PMC4209996

[B4] Asha P. K. Blouin R. T. Zaniewski R. Deutscher M. P. (1983). Ribonuclease BN: identification and partial characterization of a new tRNA processing enzyme. *Proc. Natl. Acad. Sci. U.S.A.* 80 3301–3304. 10.1073/pnas.80.11.3301 6344080 PMC394029

[B5] Ashwal-Fluss R. Meyer M. Pamudurti N. R. Ivanov A. Bartok O. Hanan M. (2014). circRNA biogenesis competes with pre-mRNA splicing. *Mol. Cell* 56 55–66. 10.1016/j.molcel.2014.08.019 25242144

[B6] Aufiero S. Reckman Y. J. Pinto Y. M. Creemers E. E. (2019). Circular RNAs open a new chapter in cardiovascular biology. *Nat. Rev. Cardiol.* 16 503–514. 30952956 10.1038/s41569-019-0185-2

[B7] Authors/Task Force M. Elliott P. M. Anastasakis A. Borger M. A. Borggrefe M. Cecchi F. (2014). 2014 ESC Guidelines on diagnosis and management of hypertrophic cardiomyopathy: the Task Force for the Diagnosis and Management of Hypertrophic Cardiomyopathy of the European Society of Cardiology (ESC). *Eur. Heart J.* 35 2733–2779. 10.1093/eurheartj/ehu284 25173338

[B8] Bao X. Zheng S. Mao S. Gu T. Liu S. Sun J. (2018). A potential risk factor of essential hypertension in case-control study: Circular RNA hsa_circ_0037911. *Biochem. Biophys. Res. Commun.* 498 789–794. 10.1016/j.bbrc.2018.03.059 29526758

[B9] Brauch K. M. Karst M. L. Herron K. J. de Andrade M. Pellikka P. A. Rodeheffer R. J. (2009). Mutations in ribonucleic acid binding protein gene cause familial dilated cardiomyopathy. *J. Am. Coll. Cardiol.* 54 930–941. 10.1016/j.jacc.2009.05.038 19712804 PMC2782634

[B10] Burd C. E. Jeck W. R. Liu Y. Sanoff H. K. Wang Z. Sharpless N. E. (2010). Expression of linear and novel circular forms of an INK4/ARF-associated non-coding RNA correlates with Atherosclerosis risk. *PLoS Genet.* 6:e1001233. 10.1371/journal.pgen.1001233 21151960 PMC2996334

[B11] Cai L. Qi B. Wu X. Peng S. Zhou G. Wei Y. (2019). Circular RNA Ttc3 regulates cardiac function after myocardial infarction by sponging miR-15b. *J. Mol. Cell Cardiol.* 130 10–22. 10.1016/j.yjmcc.2019.03.007 30876857

[B12] Capel B. Swain A. Nicolis S. Hacker A. Walter M. Koopman P. (1993). Circular transcripts of the testis-determining gene Sry in adult mouse testis. *Cell* 73 1019–1030. 10.1016/0092-8674(93)90279-y 7684656

[B13] Chen C. Y. Sarnow P. (1995). Initiation of protein synthesis by the eukaryotic translational apparatus on circular RNAs. *Science* 268 415–417. 10.1126/science.7536344 7536344

[B14] Chen J. Zou Q. Lv D. Wei Y. Raza M. A. Chen Y. (2018). Comprehensive transcriptional landscape of porcine cardiac and skeletal muscles reveals differences of aging. *Oncotarget* 9 1524–1541. 10.18632/oncotarget.23290 29416711 PMC5788579

[B15] Chen J. J. Cui L. Q. Yuan J. L. Zhang Y. Q. Sang H. J. (2017). Circular RNA WDR77 target FGF-2 to regulate vascular smooth muscle cells proliferation and migration by sponging miR-124. *Biochem. Biophys. Res. Commun.* 494 126–132. 10.1016/j.bbrc.2017.10.068 29042195

[B16] Chen X. Ba Y. Ma L. Cai X. Yin Y. Wang K. (2008). Characterization of microRNAs in serum: a novel class of biomarkers for diagnosis of cancer and other diseases. *Cell Res.* 18 997–1006. 10.1038/cr.2008.282 18766170

[B17] Cocquerelle C. Daubersies P. Majerus M. A. Kerckaert J. P. Bailleul B. (1992). Splicing with inverted order of exons occurs proximal to large introns. *EMBO J.* 11 1095–1098. 1339341 10.1002/j.1460-2075.1992.tb05148.x PMC556550

[B18] Cocquerelle C. Mascrez B. Hetuin D. Bailleul B. (1993). Mis-splicing yields circular RNA molecules. *FASEB J.* 7 155–160. 10.1096/fasebj.7.1.7678559 7678559

[B19] Conn S. J. Pillman K. A. Toubia J. Conn V. M. Salmanidis M. Phillips C. A. (2015). The RNA binding protein quaking regulates formation of circRNAs. *Cell* 160 1125–1134. 10.1016/j.cell.2015.02.014 25768908

[B20] Dang R. Y. Liu F. L. Li Y. (2017). Circular RNA hsa_circ_0010729 regulates vascular endothelial cell proliferation and apoptosis by targeting the miR-186/HIF-1alpha axis. *Biochem. Biophys. Res. Commun.* 490 104–110. 10.1016/j.bbrc.2017.05.164 28571741

[B21] Deng Y. Y. Zhang W. P. She J. Q. Zhang L. S. Chen T. Zhou J. (2016). Circular RNA related to PPAR gamma function as ceRNA of microRNA in human acute myocardial infarction. *J Am. Coll Cardiol.* 68 C51–C52. 10.1016/j.jacc.2016.07.189

[B22] Du W. W. Yang W. Chen Y. Wu Z. K. Foster F. S. Yang Z. (2017). Foxo3 circular RNA promotes cardiac senescence by modulating multiple factors associated with stress and senescence responses. *Eur. Heart J.* 38 1402–1412. 10.1093/eurheartj/ehw001 26873092

[B23] Du W. W. Yang W. Liu E. Yang Z. Dhaliwal P. Yang B. B. (2016). Foxo3 circular RNA retards cell cycle progression via forming ternary complexes with p21 and CDK2. *Nucl. Acids Res.* 44 2846–2858. 10.1093/nar/gkw027 26861625 PMC4824104

[B24] Fan X. Weng X. Zhao Y. Chen W. Gan T. Xu D. (2017). Circular RNAs in Cardiovascular Disease: An Overview. *Biomed. Res. Int.* 2017:5135781. 10.1155/2017/5135781 28210621 PMC5292166

[B25] Garikipati V. N. S. Verma S. K. Cheng Z. J. Liang D. M. Truongcao M. M. Cimini M. (2019). Circular RNA CircFndc3b modulates cardiac repair after myocardial infarction via FUS/VEGF-A axis. *Nat. Commun.* 10:4317. 10.1038/s41467-019-11777-7 PMC6754461 31541092

[B26] Geng H. H. Li R. Su Y. M. Xiao J. Pan M. Cai X. X. (2016). The circular RNA Cdr1as promotes myocardial infarction by mediating the regulation of miR-7a on its target genes expression. *PLoS One* 11:e0151753. 10.1371/journal.pone.0151753 26998750 PMC4801407

[B27] Gupta S. K. Garg A. Bar C. Chatterjee S. Foinquinos A. Milting H. (2018). Quaking inhibits doxorubicin-mediated cardiotoxicity through regulation of cardiac circular RNA expression. *Circ. Res.* 122 246–254. 10.1161/Circresaha.117.311335 29133306 PMC5771684

[B28] Hansen T. B. Jensen T. I. Clausen B. H. Bramsen J. B. Finsen B. Damgaard C. K. (2013a). Natural RNA circles function as efficient microRNA sponges. *Nature* 495 384–388. 10.1038/nature11993 23446346

[B29] Hansen T. B. Kjems J. Damgaard C. K. (2013b). Circular RNA and miR-7 in cancer. *Cancer Res.* 73 5609–5612. 24014594 10.1158/0008-5472.CAN-13-1568

[B30] Hansen T. B. Veno M. T. Damgaard C. K. Kjems J. (2016). Comparison of circular RNA prediction tools. *Nucl. Acids Res.* 44:e58. 10.1093/nar/gkv1458 26657634 PMC4824091

[B31] Holdt L. M. Stahringer A. Sass K. Pichler G. Kulak N. A. Wilfert W. (2016). Circular non-coding RNA ANRIL modulates ribosomal RNA maturation and atherosclerosis in humans. *Nat. Commun.* 7:12429. 10.1038/ncomms12429 27539542 PMC4992165

[B32] Huang S. L. Li X. Z. Zheng H. Si X. Y. Li B. Wei G. Q. (2019). Loss of super-enhancer-regulated circRNA Nfix induces cardiac regeneration after myocardial infarction in adult mice. *Circulation* 139 2857–2876. 10.1161/Circulationaha.118.038361 30947518 PMC6629176

[B33] Jakobi T. Czaja-Hasse L. F. Reinhardt R. Dieterich C. (2016). Profiling and validation of the circular RNA repertoire in adult murine hearts. *Genom. Proteom. Bioinf.* 14 216–223. 10.1016/j.gpb.2016.02.003 27132142 PMC4996846

[B34] Jeck W. R. Sharpless N. E. (2014). Detecting and characterizing circular RNAs. *Nat. Biotechnol.* 32 453–461. 10.1038/nbt.2890 24811520 PMC4121655

[B35] Jeck W. R. Sorrentino J. A. Wang K. Slevin M. K. Burd C. E. Liu J. Z. (2013). Circular RNAs are abundant, conserved, and associated with ALU repeats. *RNA* 19 141–157. 10.1261/rna.035667.112 23249747 PMC3543092

[B36] Jin Q. F. Chen Y. Y. (2019). Silencing circular RNA circ_0010729 protects human cardiomyocytes from oxygen-glucose deprivation-induced injury by up-regulating microRNA-145-5p. *Mol. Cell Biochem.* 462 185–194. 31482388 10.1007/s11010-019-03621-9

[B37] Khan M. A. Reckman Y. J. Aufiero S. van den Hoogenhof M. M. van der Made I. Beqqali A. (2016). RBM20 regulates circular RNA production from the titin gene. *Circ. Res.* 119 996–1003. 10.1161/CIRCRESAHA.116.309568 27531932

[B38] Kolakofsky D. (1976). Isolation and characterization of Sendai virus DI-RNAs. *Cell* 8 547–555. 182384 10.1016/0092-8674(76)90223-3

[B39] Kos A. Dijkema R. Arnberg A. C. van der Meide P. H. Schellekens H. (1986). The hepatitis delta (delta) virus possesses a circular RNA. *Nature* 323 558–560. 10.1038/323558a0 2429192

[B40] Kramer M. C. Liang D. Tatomer D. C. Gold B. March Z. M. Cherry S. (2015). Combinatorial control of Drosophila circular RNA expression by intronic repeats, hnRNPs, and SR proteins. *Genes Dev.* 29 2168–2182. 10.1101/gad.270421.115 26450910 PMC4617980

[B41] Lawrie C. H. Gal S. Dunlop H. M. Pushkaran B. Liggins A. P. Pulford K. (2008). Detection of elevated levels of tumour-associated microRNAs in serum of patients with diffuse large B-cell lymphoma. *Br. J. Haematol.* 141 672–675. 10.1111/j.1365-2141.2008.07077.x 18318758

[B42] Legnini I. Di Timoteo G. Rossi F. Morlando M. Briganti F. Sthandier O. (2017). Circ-ZNF609 is a circular RNA that can be translated and functions in myogenesis. *Mol. Cell* 66 22–37. 10.1016/j.molcel.2017.02.017 28344082 PMC5387670

[B43] Lei W. Feng T. Fang X. Yu Y. Yang J. Zhao Z. A. (2018). Signature of circular RNAs in human induced pluripotent stem cells and derived cardiomyocytes. *Stem. Cell Res. Ther.* 9:56. 10.1186/s13287-018-0793-5 PMC5845222 29523209

[B44] Li C. Y. Ma L. Yu B. (2017). Circular RNA hsa_circ_0003575 regulates oxLDL induced vascular endothelial cells proliferation and angiogenesis. *Biomed. Pharmacother.* 95 1514–1519. 10.1016/j.biopha.2017.09.064 28946214

[B45] Li H. Xu J. D. Fang X. H. Zhu J. N. Yang J. Pan R. (2019). Circular RNA circRNA_000203 aggravates cardiac hypertrophy via suppressing miR26b-5p and miR-140-3p binding to Gata4. *Cardiovasc. Res.* 116 1323–1334. 10.1093/cvr/cvz215 31397837 PMC7243276

[B46] Li M. Ding W. Tariq M. A. Chang W. Zhang X. Xu W. (2018). A circular transcript of ncx1 gene mediates ischemic myocardial injury by targeting miR-133a-3p. *Theranostics* 8 5855–5869. 10.7150/thno.27285 30613267 PMC6299442

[B47] Li Y. Chen B. Huang S. (2018). Identification of circRNAs for miRNA Targets by Argonaute2 RNA immunoprecipitation and luciferase screening assays. *Methods Mol. Biol.* 1724 209–218. 10.1007/978-1-4939-7562-4_17 29322452

[B48] Li Y. S. Zhang J. W. Huo C. Q. Ding N. Li J. Y. Xiao J. (2017). Dynamic organization of lncrna and circular rna regulators collectively controlled cardiac differentiation in humans. *Ebiomedicine* 24 137–146. 10.1016/j.ebiom.2017.09.015 29037607 PMC5652025

[B49] Li Z. Y. Huang C. Bao C. Chen L. Lin M. Wang X. L. (2015). Exon-intron circular RNAs regulate transcription in the nucleus. *Nat. Struct. Mol. Biol.* 22 256–264. 10.1038/nsmb.2959 25664725

[B50] Liang W. C. Wong C. W. Liang P. P. Shi M. Cao Y. Rao S. T. (2019). Translation of the circular RNA circ-catenin promotes liver cancer cell growth through activation of the Wnt pathway. *Genome Biol* 20:84. 10.1186/s13059-019-1685-4 PMC6486691 31027518

[B51] Lim T. B. Aliwarga E. Luu T. D. A. Li Y. P. Ng S. L. Annadoray L. (2019). Targeting the highly abundant circular RNA circSlc8a1 in cardiomyocytes attenuates pressure overload induced hypertrophy. *Cardiovasc Res* 115 1998–2007. 10.1093/cvr/cvz130 31114845

[B52] Lim T. B. Lavenniah A. Foo R. S. (2020). Circles in the heart and cardiovascular system. *Cardiovasc. Res.* 116 269–278. 10.1093/cvr/cvz227 31552406

[B53] Liu C. Yao M. D. Li C. P. Shan K. Yang H. Wang J. J. (2017). Silencing of circular RNA-ZNF609 ameliorates vascular endothelial dysfunction. *Theranostics* 7 2863–2877. 10.7150/thno.19353 28824721 PMC5562221

[B54] Liu L. Liu F. B. Huang M. Xie K. Xie Q. S. Liu C. H. (2019). Circular RNA ciRS-7 promotes the proliferation and metastasis of pancreatic cancer by regulating miR-7-mediated EGFR/STAT3 signaling pathway. *Hepatob. Pancreat. Dis. Int.* 18 580–586. 10.1016/j.hbpd.2019.03.003 30898507

[B55] Maesen B. Nijs J. Maessen J. Allessie M. Schotten U. (2012). Post-operative atrial fibrillation: a maze of mechanisms. *Europace* 14 159–174. 10.1093/europace/eur208 21821851 PMC3262403

[B56] Memczak S. Jens M. Elefsinioti A. Torti F. Krueger J. Rybak A. (2013). Circular RNAs are a large class of animal RNAs with regulatory potency. *Nature* 495 333–338. 10.1038/nature11928 23446348

[B57] Meng Z. Y. Chen C. Cao H. L. Wang J. Y. Shen E. (2019). Whole transcriptome sequencing reveals biologically significant RNA markers and related regulating biological pathways in cardiomyocyte hypertrophy induced by high glucose. *J. Cell Biochem.* 120 1018–1027. 10.1002/jcb.27546 30242883

[B58] Meyer K. D. Patil D. P. Zhou J. Zinoviev A. Skabkin M. A. Elemento O. (2015). 5′. UTR m(6)A promotes cap-independent translation. *Cell* 163 999–1010. 10.1016/j.cell.2015.10.012 26593424 PMC4695625

[B59] Miao R. Wang Y. Wan J. Leng D. Gong J. Li J. (2017). Microarray expression profile of circular RNAs in chronic thromboembolic pulmonary hypertension. *Medicine* 96:e7354. 10.1097/MD.0000000000007354 28682884 PMC5502157

[B60] Molkentin J. D. Lu J. R. Antos C. L. Markham B. Richardson J. Robbins J. (1998). A calcineurin-dependent transcriptional pathway for cardiac hypertrophy. *Cell* 93 215–228. 9568714 10.1016/s0092-8674(00)81573-1 PMC4459646

[B61] Ni H. Li W. Zhuge Y. Xu S. Wang Y. Chen Y. (2019). Inhibition of circHIPK3 prevents angiotensin II-induced cardiac fibrosis by sponging miR-29b-3p. *Int. J. Cardiol.* 292 188–196. 10.1016/j.ijcard.2019.04.006 30967276

[B62] Nigro J. M. Cho K. R. Fearon E. R. Kern S. E. Ruppert J. M. Oliner J. D. (1991). Scrambled exons. *Cell* 64 607–613. 10.1016/0092-8674(91)90244-s 1991322

[B63] Odiete O. Hill M. F. Sawyer D. B. (2012). Neuregulin in cardiovascular development and disease. *Circ. Res.* 111 1376–1385. 10.1161/CIRCRESAHA.112.267286 23104879 PMC3752394

[B64] Pamudurti N. R. Bartok O. Jens M. Ashwal-Fluss R. Stottmeister C. Ruhe L. (2017). Translation of CircRNAs. *Mol. Cell* 66 9–21e27. 10.1016/j.molcel.2017.02.021 28344080 PMC5387669

[B65] Pan H. Li T. Jiang Y. Pan C. Ding Y. Huang Z. (2018). Overexpression of circular RNA ciRS-7 Abrogates the tumor suppressive effect of miR-7 on gastric cancer via PTEN/PI3K/AKT signaling pathway. *J. Cell Biochem.* 119 440–446. 10.1002/jcb.26201 28608528

[B66] Pan R. Y. Liu P. Zhou H. T. Sun W. X. Song J. Shu J. (2017). Circular RNAs promote TRPM3 expression by inhibiting hsa-miR-130a-3p in coronary artery disease patients. *Oncotarget* 8 60280–60290. 10.18632/oncotarget.19941 28947970 PMC5601138

[B67] Perriman R. Ares M. (1998). Circular mRNA can direct translation of extremely long repeating-sequence proteins in vivo. *RNA* 4 1047–1054. 10.1017/S135583829898061x 9740124 PMC1369681

[B68] Rajabi M. Kassiotis C. Razeghi P. Taegtmeyer H. (2007). Return to the fetal gene program protects the stressed heart: a strong hypothesis. *Heart Fail Rev.* 12 331–343. 17516164 10.1007/s10741-007-9034-1

[B69] Rybak-Wolf A. Stottmeister C. Glazar P. Jens M. Pino N. Giusti S. (2015). Circular RNAs in the mammalian brain are highly abundant, conserved, and dynamically expressed. *Mol. Cell* 58 870–885. 10.1016/j.molcel.2015.03.027 25921068

[B70] Schroeder R. Breitenbach M. Schweyen R. J. (1983). Mitochondrial circular RNAs are absent in sporulating cells of *Saccharomyces cerevisiae*. *Nucl. Acids Res* 11 1735–1746. 10.1093/nar/11.6.1735 6188109 PMC325832

[B71] Shan K. Liu C. Liu B. H. Chen X. Dong R. Liu X. (2017). Circular noncoding RNA HIPK3 mediates retinal vascular dysfunction in diabetes mellitus. *Circulation* 136 1629–1642. 10.1161/CIRCULATIONAHA.117.029004 28860123

[B72] Siede D. Rapti K. Gorska A. A. Katus H. A. Altmuller J. Boeckel J. N. (2017). Identification of circular RNAs with host gene-independent expression in human model systems for cardiac differentiation and disease. *J. Mol. Cell Cardiol.* 109 48–56. 10.1016/j.yjmcc.2017.06.015 28676412

[B73] Sonnenschein K. Wilczek A. L. de Gonzalo-Calvo D. Pfanne A. Derda A. A. Zwadlo C. (2019). Serum circular RNAs act as blood-based biomarkers for hypertrophic obstructive cardiomyopathy. *Sci. Rep.* 9:20350. 10.1038/s41598-019-56617-2 PMC6937321 31889077

[B74] Sun Y. Jiang X. Lv Y. Liang X. Zhao B. Bian W. (2020). Circular rna expression profiles in plasma from patients with heart failure related to platelet activity. *Biomolecules* 10:187. 10.3390/biom10020187 31991759 PMC7072558

[B75] Sun Y. Zhang S. L. Yue M. M. Li Y. Bi J. Liu H. R. (2019). Angiotensin II inhibits apoptosis of mouse aortic smooth muscle cells through regulating the circNRG-1/miR-193b-5p/NRG-1 axis. *Cell. Death Dis.* 10:362. 10.1038/s41419-019-1590-5 PMC6494886 31043588

[B76] Suzuki H. Zuo Y. H. Wang J. H. Zhang M. Q. Malhotra A. Mayeda A. (2006). Characterization of RNase R-digested cellular RNA source that consists of lariat and circular RNAs from pre-mRNA splicing. *Nucl. Acids Res.* 34:e63. 10.1093/nar/gkl151 16682442 PMC1458517

[B77] Szabo L. Salzman J. (2016). Detecting circular RNAs: bioinformatic and experimental challenges. *Nat. Rev. Genet.* 17 679–692. 10.1038/nrg.2016.114 27739534 PMC5565156

[B78] Tan W. L. Lim B. T. Anene-Nzelu C. G. Ackers-Johnson M. Dashi A. See K. (2017). A landscape of circular RNA expression in the human heart. *Cardiov. Res.* 113 298–309. 10.1093/cvr/cvw250 28082450

[B79] Tang C. M. Zhang M. Huang L. Hu Z. Q. Zhu J. N. Xiao Z. (2017). CircRNA_000203 enhances the expression of fibrosis-associated genes by derepressing targets of miR-26b-5p, Col1a2 and CTGF, in cardiac fibroblasts. *Sci. Rep.* 7:40342. 10.1038/srep40342 28079129 PMC5228128

[B80] Tay Y. Rinn J. Pandolfi P. P. (2014). The multilayered complexity of ceRNA crosstalk and competition. *Nature* 505 344–352. 10.1038/nature12986 24429633 PMC4113481

[B81] van Rooij E. Marshall W. S. Olson E. N. (2008). Toward microRNA-based therapeutics for heart disease: the sense in antisense. *Circ. Res.* 103 919–928. 10.1161/CIRCRESAHA.108.183426 18948630 PMC2725407

[B82] Vausort M. Salgado-Somoza A. Zhang L. Leszek P. Scholz M. Teren A. (2016). Myocardial infarction-associated circular RNA predicting left ventricular dysfunction. *J. Am. Coll. Cardiol.* 68 1247–1248. 10.1016/j.jacc.2016.06.040 27609688

[B83] Vilades D. Martinez-Camblor P. Ferrero-Gregori A. Bar C. Lu D. C. Xiao K. (2020). Plasma circular RNA hsa_circ_0001445 and coronary artery disease: performance as a biomarker. *Faseb J.* 34 4403–4414. 10.1096/fj.201902507R 31999007

[B84] Wang K. Gan T. Y. Li N. Liu C. Y. Zhou L. Y. Gao J. N. (2017). Circular RNA mediates cardiomyocyte death via miRNA-dependent upregulation of MTP18 expression. *Cell Death Differ.* 24 1111–1120. 10.1038/cdd.2017.61 28498369 PMC5442477

[B85] Wang K. Long B. Liu F. Wang J. X. Liu C. Y. Zhao B. (2016). A circular RNA protects the heart from pathological hypertrophy and heart failure by targeting miR-223. *Eur. Heart J* 37 2602–2611. 10.1093/eurheartj/ehv713 26802132

[B86] Wang L. Shen C. Wang Y. Zou T. Zhu H. Lu X. (2019). Identification of circular RNA Hsa_circ_0001879 and Hsa_circ_0004104 as novel biomarkers for coronary artery disease. *Atherosclerosis* 286 88–96. 10.1016/j.atherosclerosis.2019.05.006 31103880

[B87] Wang S. Chen J. Y. Yu W. Q. Deng F. (2019). Circular RNA DLGAP4 ameliorates cardiomyocyte apoptosis through regulating BCL2 via targeting miR-143 in myocardial ischemia-reperfusion injury. *Int. J. Cardiol.* 279 147–147. 10.1016/j.ijcard.2018.09.023 30213603

[B88] Werfel S. Nothjunge S. Schwarzmayr T. Strom T. M. Meitinger T. Engelhardt S. (2016). Characterization of circular RNAs in human, mouse and rat hearts. *J. Mol. Cell Cardiol.* 98 103–107. 10.1016/j.yjmcc.2016.07.007 27476877

[B89] Wesselhoeft R. A. Kowalski P. S. Anderson D. G. (2018). Engineering circular RNA for potent and stable translation in eukaryotic cells. *Nat. Commun.* 9:2629. 10.1038/s41467-018-05096-6 PMC6035260 29980667

[B90] Wu J. H. Li J. Q. Liu H. Yin J. W. Zhang M. J. Yu Z. B. (2019). Circulating plasma circular RNAs as novel diagnostic biomarkers for congenital heart disease in children. *J. Clin. Lab. Anal.* 33:e22998. 10.1002/jcla.22998 31429492 PMC6868410

[B91] Wu N. Jin L. Cai J. (2017). Profiling and bioinformatics analyses reveal differential circular RNA expression in hypertensive patients. *Clin. Exp. Hypertens* 39 454–459. 10.1080/10641963.2016.1273944 28534714

[B92] Xu T. Y. Wu J. Han P. Zhao Z. M. Song X. F. (2017). Circular RNA expression profiles and features in human tissues: a study using RNA-seq data. *Bmc Genom.* 18:680 10.1186/s12864-017-4029-23 PMC5629547 28984197

[B93] Yang Y. Fan X. Mao M. Song X. Wu P. Zhang Y. (2017). Extensive translation of circular RNAs driven by N(6)-methyladenosine. *Cell Res.* 27 626–641. 10.1038/cr.2017.31 28281539 PMC5520850

[B94] Yang Y. Gao X. Zhang M. Yan S. Sun C. Xiao F. (2018). Novel role of FBXW7 circular RNA in repressing glioma tumorigenesis. *J. Natl. Cancer Inst.* 110:435. 10.1093/jnci/djx166 28903484 PMC6019044

[B95] Yang Z. G. Awan F. M. Du W. W. Zeng Y. Lyu J. Wu (2017). The circular RNA interacts with STAT3, increasing its nuclear translocation and wound repair by modulating Dnmt3a and miR-17 function. *Mol. Ther.* 25 2062–2074. 10.1016/j.ymthe.2017.05.022 28676341 PMC5589065

[B96] Yang Z. G. Guo X. B. Li G. M. Shi Y. L. Li L. P. (2016). Long noncoding RNAs as potential biomarkers in gastric cancer: opportunities and challenges. *Cancer Lett.* 371 62–70. 10.1016/j.canlet.2015.11.011 26577810

[B97] Zeng X. X. Lin W. Guo M. Z. Zou Q. (2017). A comprehensive overview and evaluation of circular RNA detection tools. *PLos Comp. Biol.* 13:e1005420. 10.1371/journal.pcbi.1005420 28594838 PMC5466358

[B98] Zeng Y. Du W. W. Wu Y. Yang Z. Awan F. M. Li X. (2017). A circular RNA binds to and activates AKT phosphorylation and nuclear localization reducing apoptosis and enhancing cardiac repair. *Theranostics* 7 3842–3855. 10.7150/thno.19764 29109781 PMC5667408

[B99] Zhang J. Xu Y. L. Xu S. Liu Y. Yu L. M. Li Z. (2018). Plasma circular RNAs, Hsa_circRNA_025016, predict postoperative atrial fibrillation after isolated off-pump coronary artery bypass grafting. *J. Am. Heart Assoc.* 7:e006642 10.1161/JAHA.117.006642

[B100] Zhang L. Zhang Y. Xue S. Ding H. Wang Y. Qi H. Z. (2020). Clinical significance of circulating microRNAs as diagnostic biomarkers for coronary artery disease. *J. Cell Mol. Med.* 24 1146–1150. 10.1111/jcmm.14802 31709737 PMC6933363

[B101] Zhang L. Zhang Y. Zhao Y. F. Wang Y. Ding H. Xue S. (2018). Circulating miRNAs as biomarkers for early diagnosis of coronary artery disease. *Expert Opin Ther Pat* 28 591–601. 10.1080/13543776.2018.1503650 30064285

[B102] Zhang Q. Sun W. X. Han J. Cheng S. Y. Yu P. Shen L. (2020). The circular RNA hsa_circ_0007623 acts as a sponge of microRNA-297 and promotes cardiac repair. *Biochem. Biophys. Res. Co.* 523 993–1000. 10.1016/j.bbrc.2019.12.116 31973814

[B103] Zhang X. O. Wang H. B. Zhang Y. Lu X. H. Chen L. L. Yang L. (2014). Complementary sequence-mediated exon circularization. *Cell* 159 134–147. 10.1016/j.cell.2014.09.001 25242744

[B104] Zhang Y. Zhang X. O. Chen T. Xiang J. F. Yin Q. F. Xing Y. H. (2013). Circular intronic long noncoding RNAs. *Mol. Cell* 51 792–806. 10.1016/j.molcel.2013.08.017 24035497

[B105] Zhao Z. Z. Li X. J. Gao C. Y. Jian D. D. Hao P. Y. Rao L. X. (2017). Peripheral blood circular RNA hsa_circ_0124644 can be used as a diagnostic biomarker of coronary artery disease. *Sci. Rep.* 7:39918. 10.1038/srep39918 28045102 PMC5206672

[B106] Zheng C. Niu H. Li M. Zhang H. Yang Z. Tian L. (2015). Cyclic RNA hsacirc000595 regulates apoptosis of aortic smooth muscle cells. *Mol. Med. Rep.* 12 6656–6662. 10.3892/mmr.2015.4264 26324352 PMC4626120

[B107] Zheng Q. P. Bao C. Y. Guo W. J. Li S. Y. Chen J. Chen B. (2016). Circular RNA profiling reveals an abundant circHIPK3 that regulates cell growth by sponging multiple miRNAs. *Nat. Commun.* 7:11215. 10.1038/ncomms11215 27050392 PMC4823868

[B108] Zhou B. Yu J. W. (2017). A novel identified circular RNA, circRNA_010567, promotes myocardial fibrosis via suppressing miR-141 by targeting TGF-beta 1. *Biochem. Biophys. Res. Commun.* 487 769–775. 10.1016/j.bbrc.2017.04.044 28412345

[B109] Zhu Y. Pan W. Yang T. Meng X. Jiang Z. Tao L. (2019). Upregulation of circular RNA CircNFIB attenuates cardiac fibrosis by sponging miR-433. *Front. Genet.* 10:564. 10.3389/fgene.2019.00564 31316543 PMC6611413

[B110] Zirkel A. Papantonis A. (2018). Detecting circular RNAs by RNA fluorescence in situ hybridization. *Methods Mol. Biol.* 1724 69–75. 10.1007/978-1-4939-7562-4_6 29322441

[B111] Zou M. Huang C. Li X. He X. Chen Y. Liao W. (2017). Circular RNA expression profile and potential function of hsa_circRNA_101238 in human thoracic aortic dissection. *Oncotarget* 8 81825–81837. 10.18632/oncotarget.18998 29137225 PMC5669851

